# Analysis of cellular water content in T cells reveals a switch from slow metabolic water gain to rapid water influx prior to cell division

**DOI:** 10.1016/j.jbc.2022.101795

**Published:** 2022-03-03

**Authors:** A. Saragovi, T. Zilberman, G. Yasur, K. Turjeman, I. Abramovich, M. Kuchersky, E. Gottlieb, Y. Barenholz, M. Berger

**Affiliations:** 1 The Lautenberg center for Immunology and Cancer Research, The Institute for Medical Research Israel-Canada, The Hebrew University Medical School, Jerusalem, Israel; 2 Department of Geochemistry, Geological Survey of Israel, Jerusalem, Israel; 3 Laboratory of Membrane and Liposome Research, Department of Biochemistry and Molecular Biology, Institute for Medical Research Israel-Canada (IMRIC), The Hebrew University-Hadassah Medical School, Jerusalem, Israel; 4 The Ruth and Bruce Rappaport, Faculty of Medicine, Technion - Israel Institute of Technology, Haifa, Israel

**Keywords:** metabolic water, metabolism, water influx, cell growth, AQP, aquaporin, CAT-IRMS, cold aqua trap-isotope ratio mass spectrometry, DMSO, dimethyl sulfoxide, EV, electronic volume, RBC, red blood cell

## Abstract

Cell growth is driven by the acquisition and synthesis of both dry biomass and water mass. In this study, we examine the increase of water mass in T cell during cell growth. We found that T-cell growth is characterized by an initial phase of slow increase in cellular water, followed by a second phase of rapid increase in water content. To study the origin of the water gain, we developed a novel methodology we call cold aqua trap-isotope ratio mass spectrometry, which allows analysis of the isotope composition of intracellular water. Applying cold aqua trap-isotope ratio mass spectrometry, we discovered that glycolysis-coupled metabolism of water accounts on average for 11 fl out of the 20 fl of water gained per cell during the initial slow phase. In addition, we show that at the end of the rapid phase before initiation of cell division, a water influx occurs, increasing the cellular water mass by threefold. Thus, we conclude that activated T cells switch from metabolizing water to rapidly taking up water from the extracellular medium prior to cell division. Our work provides a method to analyze cell water content as well as insights into the ways cells regulate their water mass.

Cell growth requires cells to accumulate mass and to increase in physical size as a prerequisite step in development and proliferation (1, 2). To grow, cells acquire and synthesize additional dry biomass; proteins, lipids, carbohydrates, and nucleic acids (3), as well as increase their water mass both as free and hydrated molecules (4, 5, 6, 7). However, how the gain of water mass is achieved, whether primarily because of metabolism or to the change in balance between influx and outflow from the external medium, remains unknown. This has been a challenging problem to study as establishing the source of additional water molecules in cells requires both the differentiation of the intracellular fraction from the background extracellular media and the tracing of the source of water molecules added to the cells. This is important because cellular water quantity affects intracellular chemistry as well as cell intracellular viscosity and mechanics.

Previous attempts to understand the regulation of size, volume, and mass in living cells have focused on changes in total cellular mass. This is best assessed using an inertial picobalance. A recent study, in which single cells were weighed using a picobalance, revealed that the mass of cells rapidly fluctuates throughout the cell cycle. This study linked these mass fluctuations to the basic cellular processes and water transport (8). However, although this study described a major leap in our ability to measure dry and water mass changes, it did not enable the direct delineation of cellular water regulation over time. Other efforts to understand cellular water hemostasis were successful in characterizing water uptake rates following osmotic shock (5). Many of these studies used diverse optical methods to establish the dilution rate of the cytoplasm by water influx. Other studies attempted to investigate hydrodynamics at the millisecond level using Raman spectroscopy following acute deuterium exposure (9). Likewise, differences in density measurements were also used to extrapolate changes in water mass during cell growth (10). However, these indirect methodologies neither trace the source of cellular water mass gains nor directly quantify water influx rates under physiological conditions. Water isotope tracing and NMR were used to investigate organs and whole-body physiological responses in mammals (11, 12) and plants (13) but not in the resolution required for analysis at the cellular level. Thus, a method to quantify in cells the correlation between volume changes and water mass gain as well as understanding the origin of water molecules gained during cell growth is lacking.

In this study, we examined the gain of water molecules in growing T cells, as a model for water mass regulation during cell growth. We found that following activation stimuli, T-cell water mass increase occurred in two distinct phases, a slow phase and a fast phase. To establish the contribution of water influx and formation of metabolic water to each of the defined growth phases, we developed a novel method based on the intracellular water isotope composition, called cold aqua trap-isotope ratio mass spectrometry (CAT-IRMS). We then applied CAT-IRMS to establish the relative contribution of water influx *versus* metabolic water in each of the defined growth phases. We found that following stimuli, T cells switch between acquisition of metabolic water and increased water influx. During T-cell slow-growth phase, most water gains result from glycolysis and other metabolic reactions. On the other hand, at the end of the late fast-growth phase and in proximity to the initiation of cell division, we identified water influx as the predominant source of cellular water gain. We conclude that during activation, T cells switch from metabolic water–based slow growth to rapid influx–driven water mass increase. The ability to measure both the rate of water gain and the isotope composition of cells' water mass is an essential tool for the development of specific aquaporin (AQP) inhibitors as well as for the study of isotope fractionation phenomena in living cells. More importantly, it provides an approach toward a better understanding of the way in which cells determine what size to grow to before dividing.

## Results

### T-cell activation is characterized by two distinct cell growth phases

To study water mass incorporation in growing cells, we focused on the well-characterized *in vitro* T-cell activation model. Primary naïve T cells are among the smallest mammalian cells, reaching approximately 7 μm in diameter (14). Stimulation of primary T cells leads to the initiation of a tightly coordinated and well-defined cell growth program leading to a significant increase in cell size (15, 16). We examined the first 24 h after stimuli, as during this period, T cells go through rapid cell growth independent of cell division (17).

**Figure 1:**
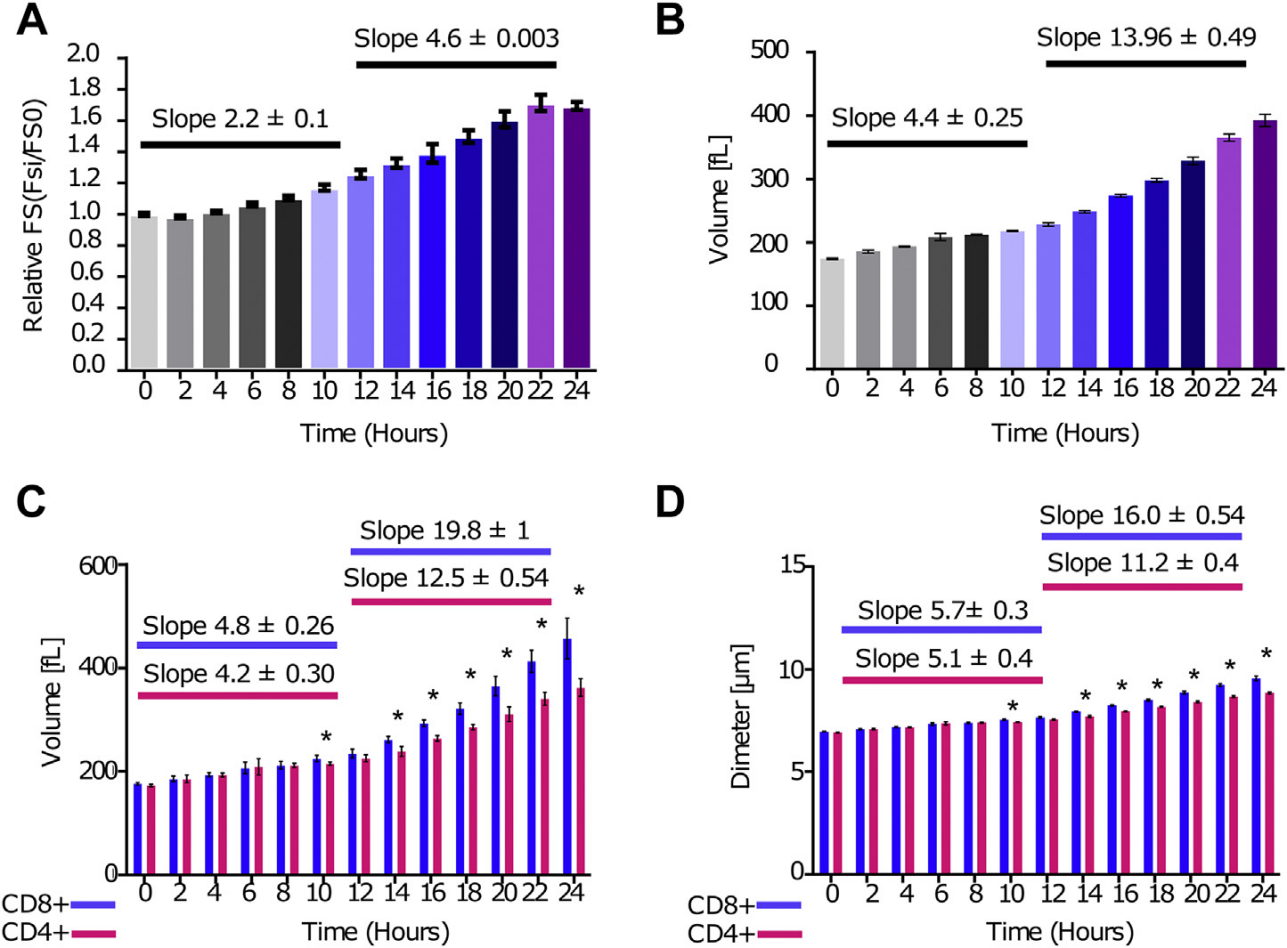
**T-cell activation is characterized by two distinct cell growth phases.***A*–*E*, mouse T cells were activated using anti-CD3 and anti-CD28 antibodies and analyzed by flow cytometer at the indicated time points after stimuli. Bar graphs show the following: (*A*) relative forward scatter (FS), the ratio between FS of activated to naïve T cells. *B*, electronic volume in femtoliter averaged per cell. *C*, electronic volume in femtoliter averaged per one CD8+ (*blue*) or CD4+ (*red*) T cell. *D*, diameter in micrometer averaged per one CD8+ (*blue*) or CD4+ (*red*) T cell. *C* and *D*, significance was established based on Mann–Whitney test; ∗<0.05. Error bars represent SEM.

To explore the dynamics of T-cell growth following stimuli, we activated primary T cells *in vitro* using a combination of anti-CD3 and anti-CD28 antibodies. T-cell size was approximated using flow cytometry at different time points after stimuli. Analysis of forward scatter signal suggested a two-phase growth dynamic; early slow-growth and late fast-growth phases (Fig. 1*A*). Forward scatter signal is a relative parameter and does not always correlate with cell size (18).To reach a more robust volume measurement, stimulated T cells were analyzed using a flow cytometer capable of detecting cell electronic volume (EV) based on changes in impendence, in accordance with the Coulter principle. In line with the forward scatter signal, EV measurements demonstrated a two-phase growth dynamic. In the first slow-growth phase, the initial 12 h following stimuli, average volume per T cell increased from 174.3 to 228.3 fl, at a rate of ∼4.4 fl/h. During the late fast-growth phase, from 12 to 24 h after stimuli, average volume per T cell increased from 228.3 to 392.5 fl, at a faster rate of ∼14 fl/h (Fig. 1*B*). Primary T cells are a heterogeneous population composed of both CD4+ and CD8+ T cells that are intrinsically altered in their proliferative responses upon activation (19). CD8+ T cells are tuned for rapid and extensive proliferation to support their role as cytotoxic cells, whereas CD4+ T cells undergo slow and limited division to support cytokine secretion and immune regulation (19). These marked differences in function may translate to variation in the pattern and/or the magnitude of growth. We therefore decided to investigate the growth rate of CD8+ and CD4+ T cells separately. Stimulated CD4+ or CD8+ T cells were analyzed using EV flow cytometry. Both T-cell types substantially accelerated cell growth at the late phase between 12 and 24 h after stimuli. Notably, CD8+ T cells accelerated their growth to a larger extent at 12 h compared with CD4+ T cells. During the late growth phase, the average CD8+ T cell increased by ∼19.8 fl per hour in comparison to ∼12.5 fl/h for the average CD4+ T cell (Fig. 1, *C* and *D*). Together, these results demonstrate that CD8+ and CD4+ T cells differ in their magnitude of growth. However, they share similar growth patterns by which at 12 h following stimuli, they switch from a slow-growth to a fast-growth program.

### T-cell growth is associated with a gradual decrease in water to total volume ratio in the early phase followed by a rapid increase in wet to total volume in the late phase

T-cell volume is the sum of both dry and water mass. To directly quantify the differences in T-cell water mass during activation, we modified a Karl Fischer titration-based protocol used in liposome content analysis (20). We reasoned that we will be able to quantify intracellular water by carefully clearing T-cell pellets from surrounding water.

**Figure 2:**
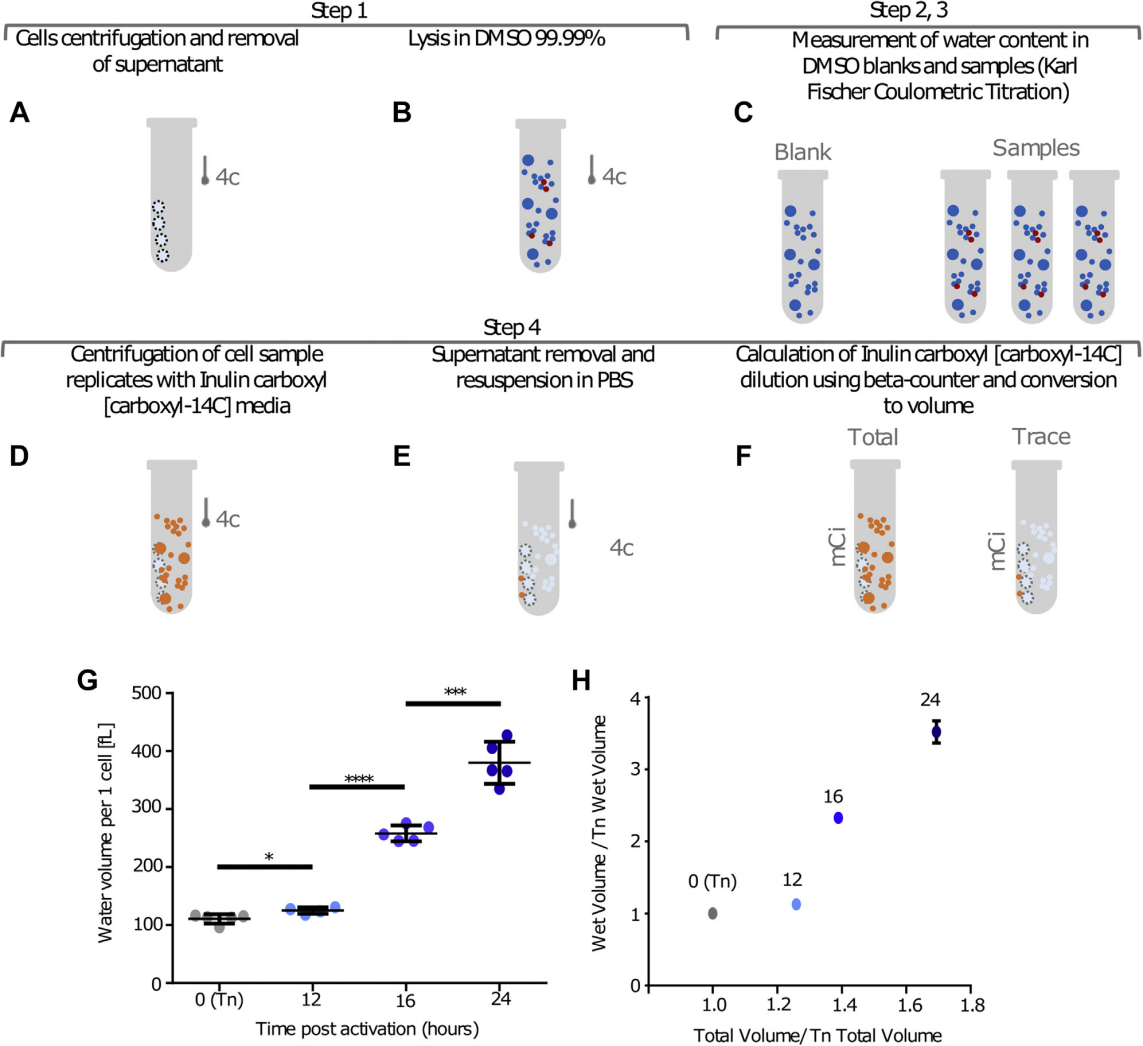
**T-cell growth is associated with a gradual decrease in wet to total volume ratio in the early phase followed by a rapid increase in wet to total volume in the late phase.***A*–*C*, schematic of the protocol used for wet volume measurements in activated T cells. *A*, tubes containing T-cell pellets were dried using narrow Whatman papers strips. *B*, T-cell pellets were dissolved in 99.99% DMSO. *C*, the water content in DMSO samples and DMSO blanks were measured using Karl Fischer coulometric titration. *D*–*F*, measurement of trace extracellular water in T-cell pellet. *D*, cell pellets were resuspended in media containing the cell-impermeable inulin carboxyl (carboxyl-^14^C). *E*, following media removal and drying, cells were resuspended in PBS. *F*, PBS with trace inulin carboxyl (carboxyl-^14^C) was then analyzed in respect to source media using beta counter to derive background volume. *G* and *H*, mouse T cells were activated using anti-CD3 and anti-CD28 antibodies, collected at the indicated time points and analyzed using the Karl Fischer coulometric titration protocol. *G*, dot plot presenting averaged water volume minus trace extracellular water, per one T cell, in femtoliter. *H*, correlation between T-cell normalized volume and wet volume at different time points (Mann–Whitney test; significance, ∗≤0.05, ∗∗∗≤0.001, and ∗∗∗∗≤0.0001). Error bars represent SEM. DMSO, dimethyl sulfoxide.

To initially measure the gross water volume in cell samples, T-cell pellets were carefully cleared from trace water and resuspended in dimethyl sulfoxide (DMSO) (Fig. 2, *A* and *B*). We then used an adjusted Karl Fischer coulometric titration protocol to quantify the water mass in the different samples as well as in DMSO blanks (Fig. 2*C*). To derive the gross water mass, the average DMSO signal (*blank*) was deducted from each sample (Table S1; Equation 1). Finally, to convert the mass to volume, we multiplied the water mass obtained with the water density coefficient, ∼1.0 (Table S1; Equation 2).

We next attempted to identify the background signal resulting from extracellular water molecules. T cells were suspended in media containing the cell-impermeable inulin carboxyl (carboxyl-^14^C) (Fig. 2*D*). Cell pellets were then carefully cleared from trace water and resuspended in PBS (Fig. 2*E*). To calculate the extracellular background level, we measured the beta radiation emitted from the PBS used to resuspend the cells (*trace*) with respect to the one from the original inulin carboxyl (carboxyl-^14^C)–based media (*source*) (Figs. 2*F* and S1*A*). To control for other possible background signal sources, we repeated the procedure with empty tubes and also measured the signal from untreated cell samples (Fig. S1*B*). Using these measurements, we could derive the indirect average extracellular water background level per sample by multiplying the dilution factor (mCi trace/mCi source) by the volume of the source sample (Fig. S1*C* and Table S1; Equation 3). By combining the background volume quantification with the growth volume measurements and accounting for the number of cells in the sample, we were able to derive the average wet volume per cell in the sample (Table S1; Equations 4 and 5).

We then applied this protocol (Fig. 2, *A*–*F*) to quantify the intracellular water mass fraction at several time points during the course of T-cell activation. Our measurements indicated that on average, naïve T cells contain ∼111 fl water per cell. Following stimuli, T-cell absolute volume increased to 125, 258, and 380 fl at 12, 16, and 24 h, respectively (Fig. 2*G*). Importantly, T-cell relative wet volume decreased at the slow-growth phase from ∼60% to ∼55% of total volume. In contrast, in the fast phase, the rapid increase in cell wet volume translated to an increase to over 95% water in respect to total cell volume (Figs. 2*H* and S1*D*). Thus, slow-growth phase of the T cells is associated with a relative decrease in cellular water quantity, whereas fast-growth phase of the T cells is associated with a substantial increase in relative wet volume. These findings suggest that the two well-defined T-cell growth phases are underlined by different water mass acquisition mechanisms.

### CAT-IRMS, a direct and robust method for quantifying and identifying the source of cell water mass gain

To identify the source of water gained by T cells during each growth phase, we developed a strategy for intracellular water tracing. Inspired by water tracing studies in plants and bacterial cells (21, 22), we designed a three-step protocol for measuring both water influx into cells as well as *de novo* metabolic water. We thought that by exposing cells for short periods to labeled media; trapping the intracellular fraction by low temperature; and analyzing the isotope ratios in the trapped media, we could detect the cellular water isotope composition and derive both the source of and the rate of water gained by the cells (Fig. S2, *A*–*F*). We named this method CAT-IRMS. To trace the entry of extracellular water to the cytoplasm, we chose to treat the cells with water labeled with heavy oxygen isotope (H_2_^18^O)-based medium (Fig. S2*A*). Alternative markers such as deuterium and tritium may diffuse from the labeled solute to various other molecules *via* proton hopping (23). Likewise, deuterium-based medium was shown to be toxic to key eukaryotic cellular process including the function of ATP synthase (24, 25). In contrast, H_2_^18^O-based labeled medium is inert and remains stable in diverse temperatures (26).

A major challenge in isolating the isotope composition of cellular water mass is to separate the intracellular fraction from the extracellular H_2_^18^O-based treatment media. We reasoned that by rapidly cooling cells to a temperature above freezing, we would be able to trap the aqueous content of cells. This is expected because of the thermodynamic effect of low temperature on the rate of osmosis across the cell membrane. Importantly, because water molecules in eukaryotic cells are locked in a tight sponge-like filament network, the reduction in energy mediated by cooling is expected to cage intracellular water molecules independent of membrane permeability (27). Thus, by cooling the cells, the isotopic composition of intracellular water could then be used to derive both the source and the rate by which cells gained water mass (Fig. S2*B*). To release the trapped intracellular water mass for isotope analysis, a measured volume of double-distilled water was added followed by several freeze–thaw cycles and a sonication protocol (Fig. S2*C*). Several control samples were collected and analyzed in parallel to cell samples to account for possible system noise. These included a sample from the labeled media following the treatment (treatment samples) for H_2_^18^O concentration measurement; a sample from the last cold wash media (background) to account for leaks and extracellular contaminants; a sample from the double-distilled water media used to lyse the cells as a baseline. Cell lysate (samples), as well as the specified control samples, was then sent for IRMS analysis (Fig. S2, *D*–*F*). Since IRMS is fed with gas, a stoichiotransfer was conducted by exposing the samples in close tubes to a gas mixture containing 99.4% helium and 0.6% carbon dioxide for 48 h at room temperature (Fig. S2*D*). This allows enough time for the oxygen in the carbon dioxide gas to equilibrate with the oxygen in the water. The gas samples containing the oxygen isotope signature were then measured by IRMS (Fig. S2*E*). To assess the minimum sample volume required for stoichiotransfer, we analyzed gas extracts following 48 h of exposure to decreasing volumes of premeasured H_2_O TW17 isotope standard. Notably, samples greater than 100 μl produced results consistent with the standard, suggesting that volumes ≥100 μl are suitable for stoichiotransfer (Fig. S3*A*). IRMS-derived ratios from the different samples were standardized in respect to the Standard Mean Ocean Water isotope ratio (Fig. S2*F*). The measurement of the ^18^O/^16^0 ratio, expressed as δ^18^O for water samples, was made following the CO_2_ equilibration method (28, 29). The results from the standardized IRMS measurements could then be used to derive the total volume of cellular H_2_^18^O in fL and the flux rate, H_2_^18^O∗min^−1^ (Table S1; Equations 6–9).

**Figure 3:**
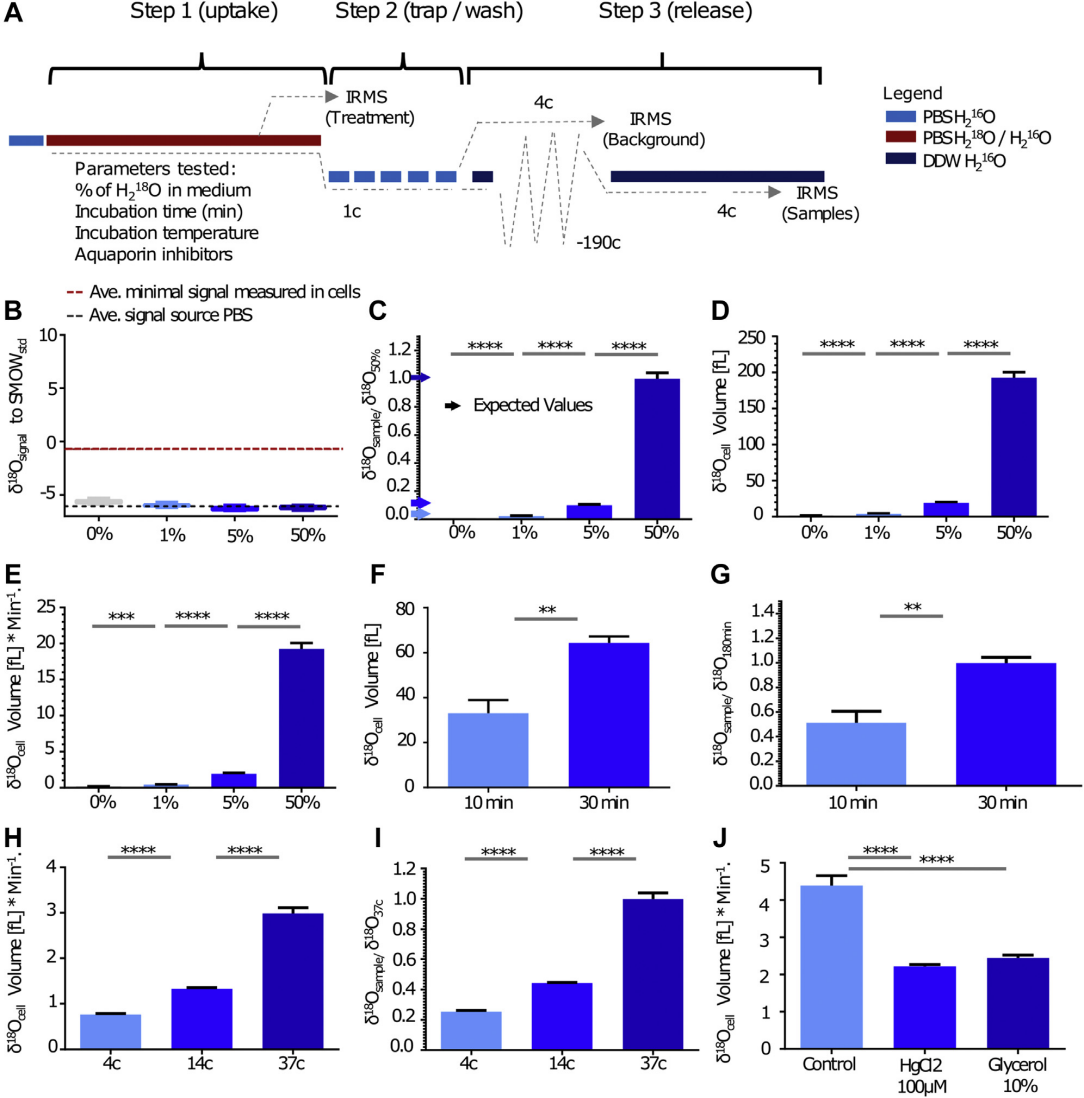
**CAT-IRMS, a direct and robust method for quantifying and identifying the source of cell water mass gain.***A*, schematic of overall CAT-IRMS calibration experiments. *B*–*E*, Hep2G cells were cultured in PBS containing the indicated percent of H_2_^18^O for 10 min. *B*, signal levels in background; δ^18^O in the wash media used in the last washing step. *Black line* represents the average signal in PBS used. *Red line* represents the average signal in the cells incubated in 1% H_2_^18^O (showed in *C*). *C*, relative δ^18^O; δ^18^O in the sample divided by the average δ^18^O in the cells that were incubated in 50% H_2_^18^O. *Arrows* represent the expected values in each concentration group. *D*, averaged H_2_^18^O volume in femtoliter per cell. *E*, H_2_^18^O volume averaged per one cell, per minute (volume of H_2_^18^O per cell divided by 10 min of incubation time). *F* and *G*, Hep2G cells were cultured in PBS containing 10% H_2_^18^O for indicated time intervals. *F*, H_2_^18^O volume averaged per one cell. *G*, H_2_^18^O of a sample relative to H_2_^18^O in the average sample incubated for 30 min. *H* and *I*, mouse peritoneal macrophages were cultured in PBS containing 50% H_2_^18^O for 10 min at the indicated temperatures. *H*, H_2_^18^O volume averaged per cell, per minute. *I*, H_2_^18^O volume in a sample relative to the average H_2_^18^O volume in samples that were incubated at 37 °C. *J*, H_2_^18^O volume averaged per cell, per minute in peritoneal macrophages incubated in PBS containing 50% H_2_^18^O for 10 min at 37 °C in the absence or the presence of the indicated aquaporin's inhibitors. (Mann–Whitney test; significance, ns > 0.05, ∗∗≤0.01, ∗∗∗≤0.001, ∗∗∗∗≤0.0001. Error bars represent SEM). CAT-IRMS, cold aqua trap-isotope ratio mass spectrometry; ns, not significant.

To test the robustness and accuracy of CAT-IRMS, we applied our methodology to a number of cell types under diverse conditions. We next examined the influence of H_2_^18^O concentration on the signal level received from cell samples. To assess the limit of detection, Hep2G cells were incubated in media containing 1%, 5%, and 50% H_2_^18^O and then analyzed *via* CAT-IRMS (Fig. 3*A*). Background measurements from the final cell wash were all comparable to the source PBS signal level and 5 permil lower than the sample signal of the cells incubated in 1% H_2_^18^O-enriched media (Figs. 3*B* and S3*B*). In contrast to background measurements, cell sample measurements produced strong signals, in proportion to the H_2_^18^O concentrations in the incubation media (Figs. 3*B* and S3*C*). To relate our results to cell number and incubation time, measurements were converted and averaged to H_2_^18^O volume in fL per cell (Fig. 3*C*) and as flux per minute (Fig. 3*D*). Under physiological conditions, the accumulation of H_2_^18^O signal in resting cells is anticipated as both growing and quiescent cells are subject to rapid fluctuation in cell density (8). Because of the concentration of H_2_^18^O, the probability of labeled water to enter the cell is higher than the probability of outflow, so resting cells are expected to accumulate a signal.

To assess the effect of incubation time on the H_2_^18^O signal, Hep2G cells were cultured in media containing 10% H_2_^18^O for 10 or 30 min and analyzed for oxygen isotope composition using CAT-IRMS. As expected, cell samples incubated for 30 min had a substantially higher H_2_^18^O signal in comparison to cell samples incubated for 10 min (Figs. 3, *F* and *G* and S3*D*). To examine the relevance of CAT-IRMS to primary cells and the effect of temperature on water flux, mouse peritoneal macrophages were cultured in PBS containing 50% H_2_^18^O for 10 min at different temperatures. In line with previous reports (13, 30, 31), signal from samples of cells incubated in 4 °C showed a fivefold signal reduction in respect to cells cultured in 37 °C (Figs. 3,*H* and *I* and S3*E*). Importantly, the reduction in the signal under low temperature indicates that the potential signal lost during the cold wash cycles step in CAT-IRMS is negligible. To evaluate whether our method can detect alterations in water flux across cell membranes, we measured H_2_^18^O accumulation in cells under AQP inhibition or hypertonic conditions. For this purpose, mouse peritoneal macrophages were incubated in media containing 50% H_2_^18^O, with or without mercury (II) chloride, an inhibitor of several AQPs (32, 33), or glycerol at concentration sufficient to induce robust osmotic gradient to drive cellular water efflux (34). As expected, CAT-IRMS analysis of samples treated with both mercury (II) chloride and glycerol showed a strong reduction in H_2_^18^O signal in respect to untreated cells (Figs. 3*J* and S3*F*). Collectively, our measurements indicate that CAT-IRMS is a robust and an accurate method, appropriate for analysis and tracing of cellular water flux, synthesis, source, and efficiency of AQP inhibitors.

### T cells switch from metabolic water gain during the slow-growth phase to water influx at the fast-growth phase

**Figure 4:**
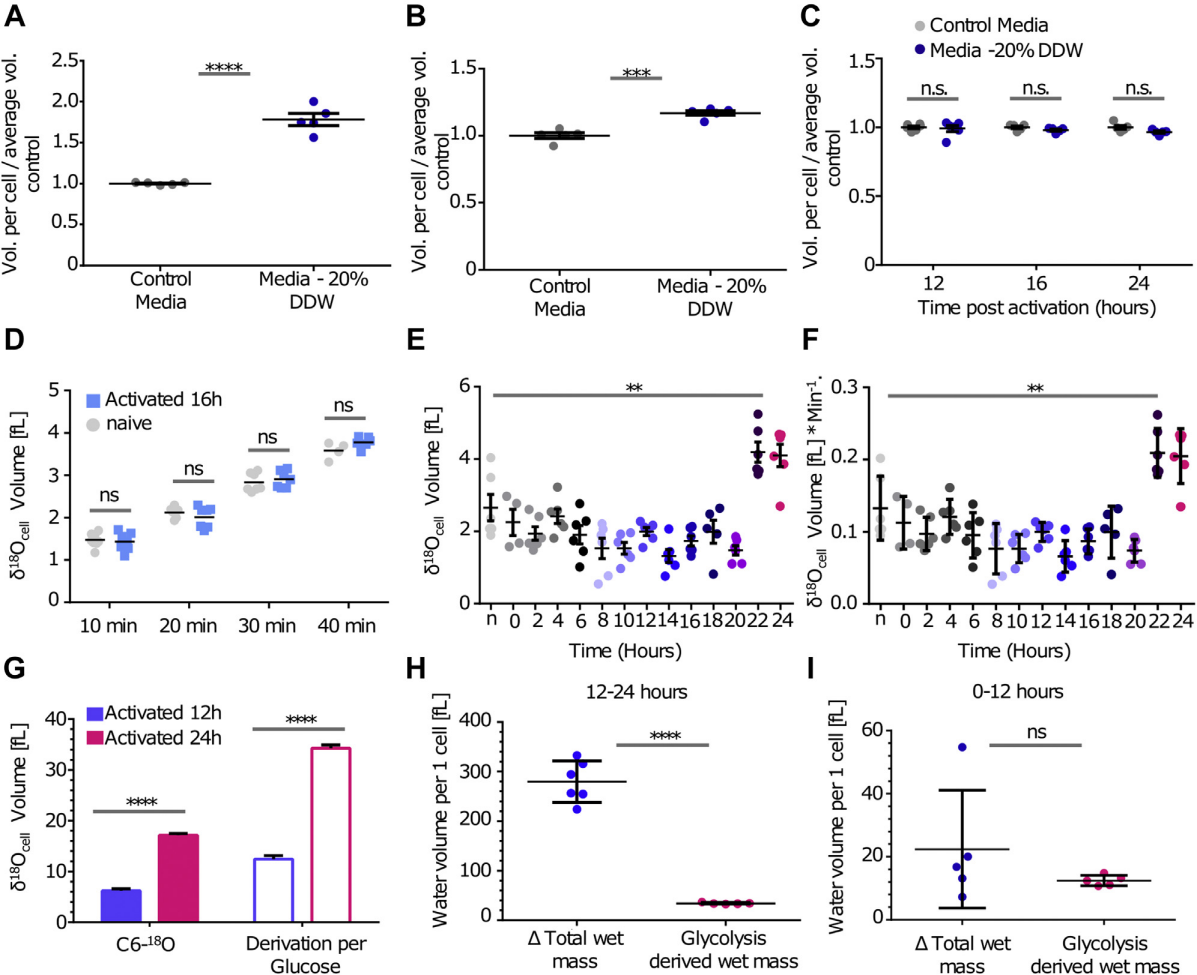
**T cells switch from metabolic water gain during the slow-growth phase to water influx at the fast-growth phase.***A*, human red blood cells were incubated for 12 h in full media (*gray*) or media containing 20% DDW (*dark blue*) and then analyzed *via* EV flow cytometry. *B*, splenocytes were incubated for 12 h in full media (*gray*) or media containing 20% DDW (*dark blue*) and then analyzed *via* EV flow cytometry (*t* test; significance, ∗ns > 0.05, ∗∗∗≤0.001, ∗∗∗∗≤0.0001; error bars represent SEM). *C*, splenocytes were activated using anti-CD3 and anti-CD28 in full media (*gray*), media containing 20% DDW (*dark blue*). Following 12, 16, and 24 h, T-cell size was compared *via* EV low cytometry analysis. (Two-way ANOVA test). *D*, H_2_^18^O volume averaged per cell for mouse naïve or 16 h stimulated T cells that were cultured in a medium containing 50% H_2_^18^O for indicated time intervals. *E* and *F*, T-cell samples were collected at the indicated time points following stimulation, incubated with 50% H_2_^18^O for 10 min, and analyzed by CAT-IRMS. *E*, H_2_^18^O volume averaged per cell. *F*, H_2_^18^O volume averaged per cell per minute. *G*–*I*, T cells were stimulated in medium containing C-[6-^18^O]glucose for 12 or 24 h. *G*, H_2_^18^O volume measured using CAT-IRMS. Empty frames represent derived values for each glucose molecule (two water molecules per one glucose molecule). Calculated glycolysis derived *de novo* water biogenesis in respect to total change in water volume. *H*, during the first 12 h or (*I*) 24 h of T-cell activation, averaged per cell. (Mann–Whitney test; significance, ns > 0.05, ∗∗≤0.01, ∗∗∗∗≤0.0001. Error bars represent SEM). CAT-IRMS, cold aqua trap-isotope ratio mass spectrometry; DDW, double-distilled water; EV, electronic volume; ns, not significant.

The sponge model suggests that in eukaryote cells with dense cytoskeleton, the regulation of intracellular water is not a simple function of extracellular media tonicity (7, 27). We therefore examined how changes in media tonicity affect naïve and activated T-cell volume in respect to red blood cells (RBCs). Incubation of RBCs and resting naïve T cells in hypotonic media led to a significant inflation in cell volume (Fig. 4, *A* and *B*). In contrast, T cells activated in hypotonic media for 12, 16, and 24 h did not demonstrate a significant increase in volume with respect to cells in normal media (Fig. 4*C*). These results suggest that as opposed to quiescent naïve T cells and RBCs, activated T cells may have the capacity to maintain regulated volume decrease (35) over time. This alteration can be attributed to augmented metabolism and/or cytoskeleton reprogramming upheld by activated T cells that can serve as compensatory mechanisms for the influx of water over time. Overall, these findings indicate that cellular water mass growth reflects a tightly regulated water flux mechanism, linked to membrane permeability, ion transport, the ability of the cytoplasm to absorb water, and biogenesis of *de novo* water molecules by cellular metabolism. To directly test whether stimulated T cells increase the influx of water molecules during growth, we applied CAT-IRMS to measure water entry from extracellular media after stimulation. Naïve and activated T cells, 16 h after stimuli, were cultured for 10, 20, 30, and 40 min in media containing 50% H_2_^18^O. Interestingly, IRMS measurements detected no significant differences in cellular H_2_^18^O content between the naïve, quiescent, and stimulated growing cells (Figs. 4*D* and S4*A*). To quantify water influx across the first 24 h of activation, stimulated T cells were treated with media containing 50% H_2_^18^O at 13 time points after stimuli. Consistent with our previous results, IRMS measurements detected no significant increase in water uptake by activated cells in the first 22 h after stimulation. At the late activation period, 22 to 24 h after stimulation, a twofold increase in the rate of water uptake was detected, suggesting an additional switch in T-cell hydrodynamics around 22 h after stimuli (Figs. 4, *E* and *F* and S4*B*).

Following stimulation, T cells rewire their metabolism to support cellular growth. We reasoned that *de novo* water biogenesis might contribute to cellular water mass as a byproduct of these metabolic and anabolic reactions. A hallmark of T-cell metabolic alteration following activation is a marked increase in glycolysis (36). At the ninth step of the glycolysis process, the enzyme enolase catalyzes the conversion of two phosphoglycerate to phosphoenolpyruvate. The hydroxyl groups originated from the carbons in position 6 or 3 in the original glucose molecule are then donated to generate water. To investigate the contribution of glycolysis to intracellular water, we first measured the quantity of glucose consumed by T cells during activation *via* targeted metabolic analysis. At the first 24 h after stimuli, we observed more than 40% reduction in media glucose concentration relative to source (Fig. S4*C*). Given the number of cells, the glucose consumed may account for *de novo* biosynthesis of 36 fl of water molecules per cell in total or 1.5 fl per hour.

To directly measure glycolysis-coupled *de novo* water biogenesis in activated T cells, we used CAT-IRMS to measure the production of H_2_^18^O from d-[6 ^18^O]-glucose. Stimulated T cells cultured for 12 and 24 h produced strong H_2_^18^O signal reaching ∼5 and ∼15 fl for the average cell, respectively (Figs. 4*G* and S4*D*). To derive the contribution of *de novo* water biosynthesis to cellular water mass and since d-[6 ^18^O]-glucose labeling accounts for only one of two water molecules generated, we calculated the final values based on a coefficient of 2 (Fig. 4*G*, *empty frames*). To compare the water generated by glycolysis to the wet volume gained at each growth phase, we analyzed our measurements relative to the water gained in the two distinct growth phases. Our analysis indicated that glycolysis-derived *de novo* water biogenesis has a marginal contribution to wet volume gains during the fast-growth phase (Fig. 4*H*). In contrast, at the slow phase, glycolysis-derived *de novo* water biogenesis accounts for the majority of the increase in water volume gained during the first 12 h (Fig. 4*I*). Furthermore, since water is a byproduct of mitochondrial respiration as well as protein and RNA synthesis, H_2_^18^O measurements vastly underestimate the total intracellular *de novo* water synthesized during T-cell activation. Taken together, our findings provide compiling evidence that during activation, T cells switch from a slow metabolism-coupled water gain phase to fast and flux-based water mass growth.

## Discussion

Cell growth is a critical step in the development and proliferation of cells. It has not been clear how water molecules are attained during cell growth. The abundance of water in the extracellular fluid may suggest that growing cells obtain additional water mass by increasing the net influx of water. However, such a mechanism would require growing cells to continually regulate water fluxes with respect to the increase of intracellular osmolality. An intriguing alternative is that cells attain water molecules by *de novo* biosynthesis, as a byproduct of metabolic reactions, such as glycolysis, respiration, and protein synthesis. Thus, the increase of water mass in growing cells may be coupled to the underlying metabolism of cell growth.

CAT-IRMS, an ^18^O-based method developed in our laboratory, allows direct measurement of the rate of water influx. Analyzing the isotopic composition of intracellular water is useful not only to trace the source of water flux but also for identifying *de novo* water generated as a byproduct of metabolism. An additional advantage of the method is its capability to record over a time scale of hours short-term instantaneous water influx events, which are hard to detect with current methods. Furthermore, we have demonstrated that CAT-IRMS could be applied to different cell lines and primary cells and be used as a standard to trace intracellular water in multiple applications. Using CAT-IRMS on activated T cells, we identified three different dominant cellular water gain mechanisms (model provided in Fig. S5*A*). Following activation and during the slow-growth phase, T cells acquire water as a byproduct of glycolysis and other metabolic reactions. In contrast, at the late fast-growth phase, the increase in water could not be explained by either *de novo* water biogenesis or increased influx. Thus, our observations suggest that during the fast-growth phase, T cells may increase the overall water volume by decreasing water outflow while keeping influx at the basal level. Finally, as cells approach division, we observed a twofold increase in the rate of water influx. Our study reveals that the gain of water in the T cells early slow-growth phase is coupled to the rate of specific metabolic reactions such as glycolysis. Thus, alterations in T-cell metabolic program during activation may lead to differences in the dry-to-water mass ratio between cells. Future work may focus on improving the method accuracy and accessibility. Applying the method for detecting water outflux remains challenging and requires the development of alternative protocols to the one presented in this study. Likewise, it remains unknown whether the method could be used to detect water influx or the generation of metabolic water *in vivo*. Finally, reducing the dependency of the method on IRMS, which is not commonly available, may improve the accessibility of the method to the research community. Our work opens a path for a better understanding of cellular growth and for studying the way cells determine what size to grow to before dividing.

## Experimental procedures

### Mice

The C57BL/6J (wildtype) mice were from The Jackson Laboratory. All mice used in this study maintained and bred under specific pathogen-free conditions in the Hebrew University animal facilities according to Institutional Animal Care and Use Committee regulations. All experiments performed in mice were approved by the Institutional Animal Care and Use Committee at the Hebrew University (National Institutes of Health approval number: OPRR-A01-5011). All mice were maintained on the C57BL/6J background and used for experiments at 8 to 12 weeks of age.

### Human samples

Human blood samples were obtained *via* Shaare Zedek Medical Center Jerusalem. All experiments involved human blood were approved by the Shaare Zedek Medical Center Helsinki review board (Helsinki Committee approval number: 143/14).

### T-cell isolation

CD8+ or total T cells were isolated from spleens with an EasySep T Cell Isolation Kit according to the manufacturer’s instructions (STEMCELL Technologies).

### Peritoneal macrophage culture

Residential peritoneal macrophages were directly collected from anesthetized mice by lavage with 5 ml PBS using a 5 ml syringe with 19G needle. When elicited macrophages were needed, the mice were injected intraperitoneally with 1 ml 4% thioglycollate at least 36 h in advance. Then, 5 × 105 erythrocyte-depleted lavage-derived cells were plated on a tissue culture treated polystyrene 24-well plates (Corning).

### *In vitro* T-cell proliferation assay

Primary isolated T cells or CD8+ were activated in 96-flat-well plates (1 × 10^6^ cells per well) coated with anti-CD3ε (6 μg/ml) and anti-CD28 (6 μg/ml).

### Flow cytometry and EV

Cells were stained with various conjugated monoclonal antibodies against cell-surface markers in fluorescent-activated cell sorting buffer (PBS containing 1% fetal bovine serum) for 30 min at 4 °C. Stained cells were assayed by Gallios flow cytometer with Kaluza software (Beckman Coulter) or iCyt Eclipse (Sony) and analyzed by FACS Express 6 (De Novo Software).

### Karl Fischer titration

Isolated naïve T cells were centrifuged in 1.7 ml tubes. Following careful removal of all visual liquids, pellets were resuspended in DMSO (Sigma; 99.99%). Samples were measured in 831 KF Coulometer (Metrohm). For calculation of background noise, blank DMSO measurements were performed. To calculate the noise from water leftovers, 10 samples were suspended in PBS containing inulin carboxyl (carboxyl-^14^C). Samples were then centrifuged, and PBS was removed. Cells were resuspended in 1 ml PBS and inulin carboxyl (carboxyl-^14^C) radioactivity was measured to evaluate dilution in respect to standard.

### Targeted metabolic analysis

CD8+ T cells were cultured in anti-CD3/anti-CD28-coated 96-well plate (1 million cells/well), suspended in RPMI supplemented with 10% dialyzed fetal bovine serum and 100 μM alanine. Following 24 h, activated cells were then extracted for metabolomics LC–MS analysis.

The following were the medium extracts used: 20 μl culture medium was added to 980 μl of a cold extraction solution (−20 °C) composed of methanol, acetonitrile, and water (5:3:2). Medium extracts were centrifuged (10 min at 16,000*g*) to remove insoluble material, and the supernatant was collected for LC–MS analysis. Metabolomics data were normalized to protein concentrations using a modified Lowry protein assay.

LC–MS metabolomics analysis was performed as described previously (37). Briefly, Thermo Ultimate 3000 HPLC system coupled to Q-Exactive Orbitrap Mass Spectrometer (Thermo Fisher Scientific) was used with a resolution of 35,000 at 200 mass/charge ratio (*m/z*), electrospray ionization, and polarity switching mode to enable both positive and negative ions across a mass range of 67 to 1000 *m/z*. HPLC setup consisted ZIC-pHILIC column (SeQuant; 150 mm × 2.1 mm, 5 μm; Merck), with a ZIC-pHILIC guard column (SeQuant; 20 mm × 2.1 mm). About 5 μl of biological extracts was injected, and the compounds were separated with mobile phase gradient of 15 min, starting at 20% aqueous (20 mM ammonium carbonate adjusted to pH 2 with 0.1% of 25% ammonium hydroxide) and 80% organic (acetonitrile) and terminated with 20% acetonitrile. Flow rate and column temperature were maintained at 0.2 ml/min and 45 °C, respectively, for a total run time of 27 min. All metabolites were detected using mass accuracy below 5 ppm. Thermo Xcalibur was used for data acquisition. TraceFinder 4.1 (Thermo Fisher Scientific) was used for analysis. Peak areas of metabolites were determined by using the exact mass of the singly charged ions. The retention time of metabolites was predetermined on the pHILIC column by analyzing an in-house mass spectrometry metabolite library that was built by running commercially available standards.

### IRMS

For the measurement of δ^18^O water, clean vacuum vessels were flushed with a gas mixture of helium (99.6%) and CO_2_ (0.4%) for 10 min to remove the original atmosphere. After flushing, 0.7 cm^3^ of the sampled water was injected to the vessels and left to equilibrate with the CO_2_ gas for 48 h at 25 °C. Oxygen isotopes were measured using a Finnigan Gas Bench II extraction system attached to a ThermoFinnigan Delta PLUS XP continuous flow mass spectrometer. All oxygen isotopic measurements were done in duplicate and reported relative to Vienna Standard Mean Ocean Water. Four-well calibrated internal laboratory standards were used for calibration, and a standard was measured every eight water samples.

### Statistical analysis

The statistical significance of differences was determined by the two-tailed Student's *t* test. Differences with a *p* value of less than 0.05 were considered statistically significant. GraphPad Prism software (GraphPad Software, Inc) was used for analysis.

## Data availability

The authors confirm that the data supporting the findings of this study are available within the article and its supporting information.

## Supporting information

This article contains supporting information.

## Conflict of interest

The authors declare that they have no conflicts of interest with the contents of this article.
