# Supplementary material for: Analysis of cellular water content in T cells reveals a switch from slow metabolic water gain to rapid water influx prior to cell division
Source: J Biol Chem. 2022 Mar 3;298(4):101795. doi: 10.1016/j.jbc.2022.101795 (PMC9034303; doi:10.1016/j.jbc.2022.101795)
Supplement: Supplementary Materials [file mmc1.docx]

**Analysis of cellular water content in T cells reveals a switch from slow metabolic water gain to rapid water influx prior to cell division**

Saragovi A^*^, Zilberman T, Yasur G, Turjeman K, Abramovich I, Kuchersky M, Gottlieb E, Barenholz Y, Berger M^*^

* For correspondence: Michael Berger, [michaelb@ekmd.huji.ac.il](mailto:michaelb@ekmd.huji.ac.il); Amijai Saragovi, [amijaisar@gmail.com](mailto:amijaisar@gmail.com)

**Supplementary Materials**

| 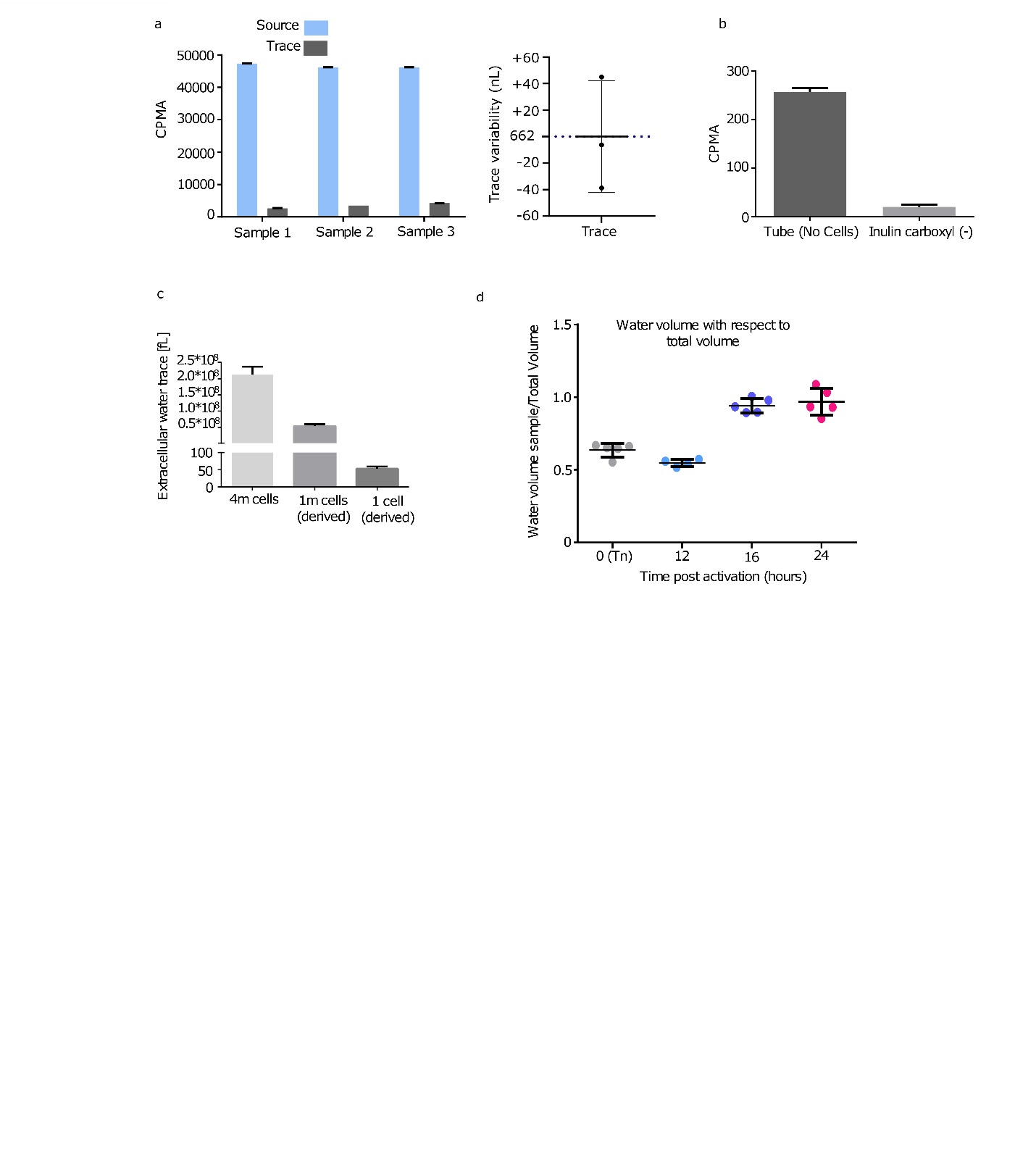 |
| --- |
| **Figure S1: Measurement of trace extracellular water for wet water measurement a.** Sample beta counter measurements of source and trace samples as described in Figure 2f (left panel). Dot plot graph demonstrating the effect of variability in trace water measured on total growth water measurment sample (right panel). **b.** Beta counter measurements of: Tube (No Cells) - Source Inulin‐Carboxyl‐^14^C media was added to empty tubes. Tubes were then dried using thin Whatman strips. Beta counter signal represents the tube background signal (signal independent of cells). Inulin carboxyl (-) -T cell samples in 100ul PBS without Inulin‐Carboxyl‐^14^C (cell background signal)**. c.** Trace extracellular background signal calculated for different number of T cells **d.** Water volume in respect to the total volume averaged per one T cell at each time point. (Error bars represent s.e.m.). |

| 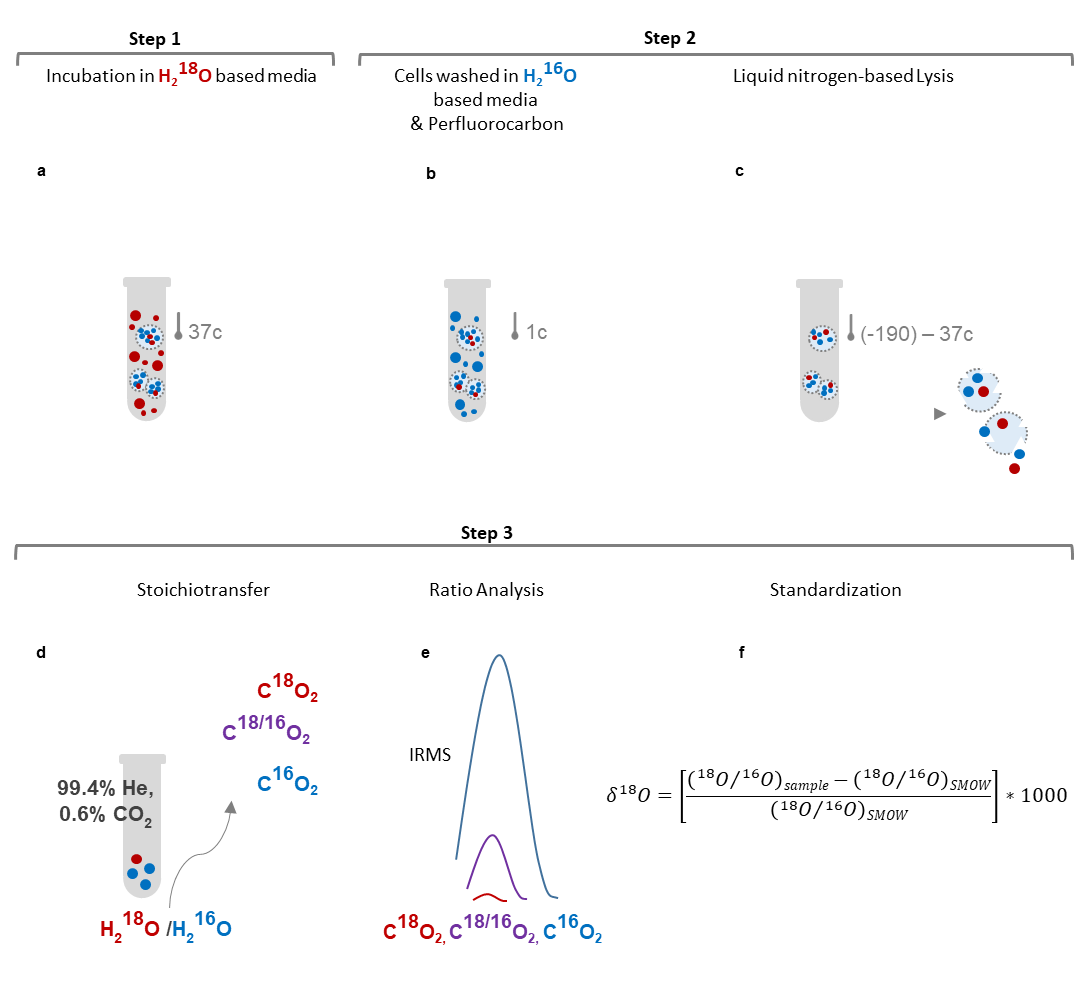 |
| --- |
| **Figure S2: Schematic steps of Cellular Aqua Trap – Isotope Ratio Mass Spectrometry (CAT-IRMS) protocol -** Step 1 – **a** Cells are incubated in PBS or culture medium containing different concentrations of H_2_^18^O at 37c for an appropriate time intervals. - Step 2 -: **b** Following the incubation, cell samples are rapidly cooled to 1°C and placed on ice in cold room. Cell incubation media is then collected (source). Cells pellets are next washed 5 times with ice cold normal PBS. **c** To release the intracellular water trapped within the cells, 400 µl DDW are then added to the cell pellets followed by 3 cycles of freeze/thaw using liquid nitrogen and sonication. Cell lysates are then collected and sent for IRMS analysis for ^18^O/^16^O ratio measurements. Media from steps 1 (source) and 2 (background) are also collected and sent to IRMS **-** Step 3:- **d** For IRMS analysis, samples were injected into sealed glass tubes filled with 99.4% He, 0.6% CO_2_  for 48 hours for stoichiometric transfer of ^18^O or ^16^O to CO_2_ **e** Gas mixture from each tube is then loaded into the IRMS for measurement **f** ^18^O volume (δ^18^O) is extrapolated by comparing to international H_2_^18^O abundance in ocean water using the presented formula (f). *SMOW*: Standard Mean Ocean Water. |

| 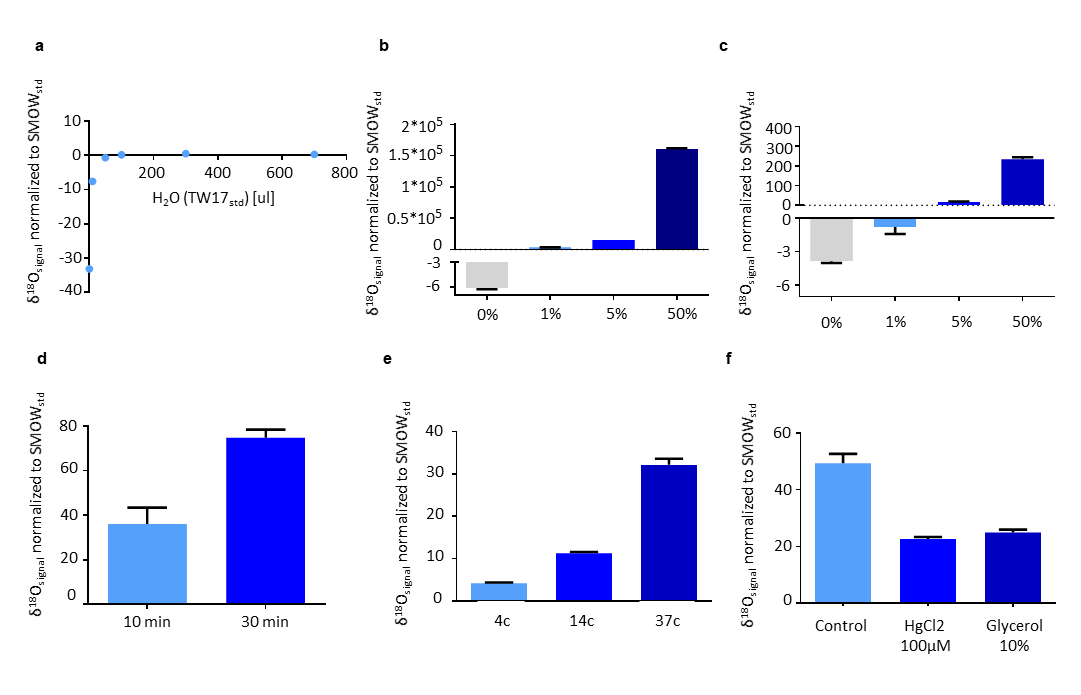 |
| --- |
| **Figure S3: Calibration of CAT-IRMS a** Measurement of minimum volume required for stoichiometric transfer in δ^18^O signal normalized to SMOW b-c. Hep2G cells were cultured in PBS containing the indicated % of H_2_^18^O for 10 minutes. **b** δ^18^O in the source media used to incubate the cells. Bars represent δ^18^O signal normalized to SMOW. **c** Cell samples δ^18^O signal normalized to SMOW. **d** Hep2G cells were cultured in PBS containing 10% H_2_^18^O for indicated time intervals. Bars represent δ^18^O signal normalized to SMOW **e** Mouse peritoneal macrophages were cultured in PBS containing 50% H_2_^18^O for 10 minutes at the indicated temperatures. Bars represent δ^18^O signal normalized to SMOW **f** δ^18^O signal normalized to SMOW in peritoneal macrophages incubated in PBS containing 50% H_2_^18^O for 10 minutes at 37°C in the absence or presence of the indicated aquaporin inhibitors. (Error bars represent s.e.m.). |

| 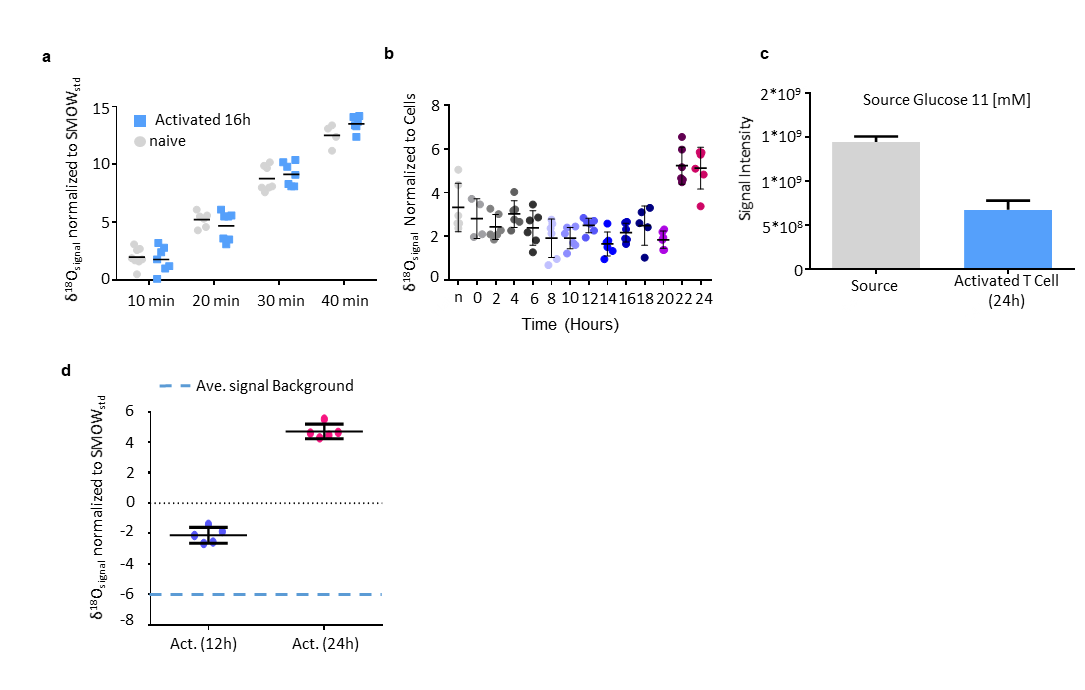 |
| --- |
| **Figure S4: T cells growth is propagated by three distinct water mass gain states. a** δ^18^O signal normalized to SMOW in samples from mouse naïve or 16 h stimulated T cells that were cultured in a medium containing 50% H_2_^18^O for indicated time intervals. **b** T cell samples were collected at the indicated time points following stimulation, incubated with 50% H_2_^18^O for 10 minutes and analyzed by CAT-IRMS. Dots represent δ^18^O signal normalized to SMOW **c** Glucose levels as signal intensity in growing media of activated T cells for 24 hours relative to glucose levels in the source media. Glucose levels were measured using LC-MS-MS. **d** Naïve or stimulated T cells were cultured in medium containing D-[6-^18^O]glucose for 12 or 24 hours. Samples were then measured using CAT-IRMS. Dots represent δ^18^O signal normalized to SMOW. (Error bars represent s.e.m.). |

| 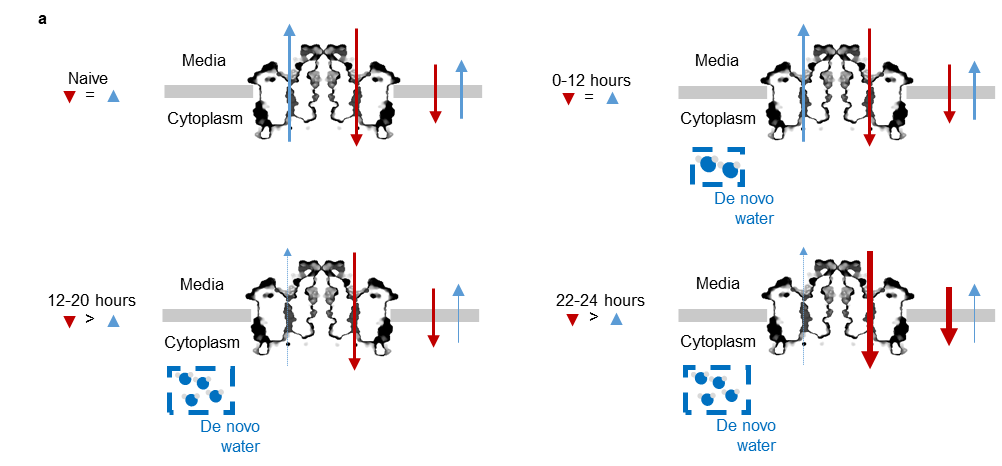 |
| --- |
| **Figure S5: T cells growth is propagated by three distinct water mass gain states.** A model representing the different states of cellular water mass gain during T cell activation. |

| **Table S1: Equations used in the study** |
| --- |
| 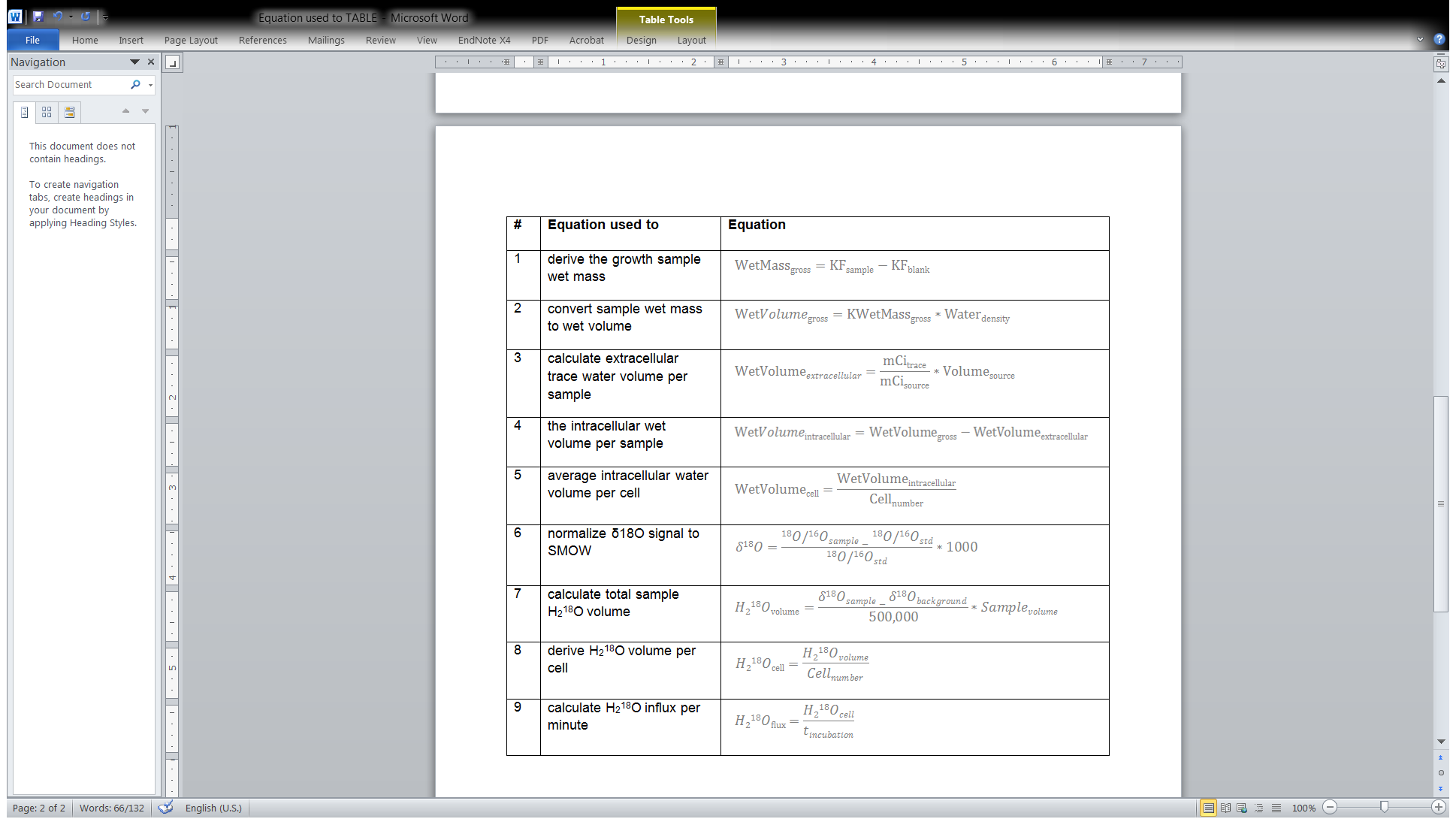 |
