# Supplementary material for: Analysis of cellular water content in T cells reveals a switch from slow metabolic water gain to rapid water influx prior to cell division
Source: J Biol Chem. 2022 Mar 3;298(4):101795. doi: 10.1016/j.jbc.2022.101795 (PMC9034303; doi:10.1016/j.jbc.2022.101795)
Supplement: Table S1 [file mmc2.pdf]

#	Equation used to	Equation
1	derive the growth sample wet mass	$\mathrm{WetMass_{gross}} = \mathrm{KF_{sample}} - \mathrm{KF_{blank}}$
2	convert sample wet mass to wet volume	$\mathrm{Wet}Volume_{\mathrm{gross}} = \mathrm{KWetMass_{gross}} * \mathrm{Water_{density}}$
3	calculate extracellular trace water volume per sample	$\mathrm{WetVolume}_{extracellular} = \frac{\mathrm{mCi_{trace}}}{\mathrm{mCi_{source}}} * \mathrm{Volume_{source}}$
4	the intracellular wet volume per sample	$\mathrm{Wet}Volume_{\mathrm{intracellular}} = \mathrm{WetVolume_{gross}} - \mathrm{WetVolume_{extracellular}}$
5	average intracellular water volume per cell	$\mathrm{WetVolume_{cell}} = \frac{\mathrm{WetVolume_{intracellular}}}{\mathrm{Cell_{number}}}$
6	normalize δ18O signal to SMOW	$\delta^{18}O = \frac{{}^{18}O/{}^{16}O_{sample} - {}^{18}O/{}^{16}O_{std}}{{}^{18}O/{}^{16}O_{std}} * 1000$
7	calculate total sample $H_2{}^{18}O$ volume	$H_2{}^{18}O_{\mathrm{volume}} = \frac{\delta^{18}O_{sample} - \delta^{18}O_{background}}{500{,}000} * Sample_{volume}$
8	derive $H_2{}^{18}O$ volume per cell	$H_2{}^{18}O_{\mathrm{cell}} = \frac{H_2{}^{18}O_{volume}}{Cell_{number}}$
9	calculate $H_2{}^{18}O$ influx per minute	$H_2{}^{18}O_{\mathrm{flux}} = \frac{H_2{}^{18}O_{cell}}{t_{incubation}}$
